# Discovery of a novel lactate dehydrogenase tetramerization domain using epitope mapping and peptides

**DOI:** 10.1016/j.jbc.2021.100422

**Published:** 2021-02-17

**Authors:** Léopold Thabault, Maxime Liberelle, Katarina Koruza, Esra Yildiz, Nicolas Joudiou, Joris Messens, Lucie Brisson, Johan Wouters, Pierre Sonveaux, Raphaël Frédérick

**Affiliations:** 1 Louvain Drug Research Institute (LDRI), Université catholique de Louvain (UCLouvain), Brussels, Belgium; 2 Pole of Pharmacology and Therapeutics, Institut de Recherche Expérimentale et Clinique (IREC), Université catholique de Louvain (UCLouvain), Brussels, Belgium; 3 VIB-VUB Center for Structural Biology, Brussels, Belgium; 4 Redox Signaling Lab, Brussels Center for Redox Biology, Brussels, Belgium; 5 Structural Biology Brussels, Vrije Universiteit Brussel, Brussels, Belgium; 6 Nuclear and Electron Spin Technologies, Louvain Drug Research Institute (LDRI), Université catholique de Louvain (UCLouvain), Brussels, Belgium; 7 Inserm UMR1069, Nutrition, Growth and Cancer, University of Tours, Tours, France; 8 NARILIS, Department of Chemistry, UNamur, University of Namur, Namur, Belgium

**Keywords:** cancer, biophysics, oligomerization, protein–protein interaction, NMR, WaterLOGSY, mass photometry, microscale thermophoresis, nanoDSF, lactate dehydrogenases, disruptors, LDH, lactate dehydrogenase, LDH-1, lactate dehydrogenase heart isozyme homotetramer, LDH-5, lactate dehydrogenase muscle isozyme homotetramer, LDH-H, lactate dehydrogenase heart isozyme, LDH-Htr, truncated dimeric version of LDH, LDH-M, lactate dehydrogenase muscle isozyme, MOE, Molecular Operating Environment, MP, mass photometry, MST, microscale thermophoresis, nanoDSF, nanoscale differential scanning fluorimetry, WaterLOGSY, water–ligand observed *via* gradient spectroscopy

## Abstract

Despite being initially regarded as a metabolic waste product, lactate is now considered to serve as a primary fuel for the tricarboxylic acid cycle in cancer cells. At the core of lactate metabolism, lactate dehydrogenases (LDHs) catalyze the interconversion of lactate to pyruvate and as such represent promising targets in cancer therapy. However, direct inhibition of the LDH active site is challenging from physicochemical and selectivity standpoints. However, LDHs are obligate tetramers. Thus, targeting the LDH tetrameric interface has emerged as an appealing strategy. In this work, we examine a dimeric construct of truncated human LDH to search for new druggable sites. We report the identification and characterization of a new cluster of interactions in the LDH tetrameric interface. Using nanoscale differential scanning fluorimetry, chemical denaturation, and mass photometry, we identified several residues (E62, D65, L71, and F72) essential for LDH tetrameric stability. Moreover, we report a family of peptide ligands based on this cluster of interactions. We next demonstrated these ligands to destabilize tetrameric LDHs through binding to this new tetrameric interface using nanoscale differential scanning fluorimetry, NMR water–ligand observed *via* gradient spectroscopy, and microscale thermophoresis. Altogether, this work provides new insights on the LDH tetrameric interface as well as valuable pharmacological tools for the development of LDH tetramer disruptors.

Dysregulation of glucose metabolism is a common feature of most cancer cells (1). The elevated glycolytic flux in cancer cells has two origins: adaptation to hypoxia (anaerobic glycolysis) and adaptation to high proliferation rates (aerobic glycolysis, also known as the “Warburg effect”) (2). This higher glycolytic flux provides cancer cells with the energy and biomass essential for the sustainment of their anabolic growth. At the end of the glycolytic pathway stands the reduction of pyruvate to lactate catalyzed by the lactate dehydrogenase (LDH) family.

While lactate has long been considered as a mere byproduct of glycolysis, it is now regarded as a potential purpose of accelerated glycolysis in cancer, in the light of the numerous benefits it provides to tumor growth (3). Elevation of lactate production indeed promotes several phenomena, such as angiogenesis (4, 5, 6), invasiveness (7, 8), commensalism (9), inflammation (10, 11), as well as redox homeostasis (12). Lactate metabolism further establishes a metabolic symbiosis between oxidative cancer cells that use lactate preferentially to glucose as a fuel and glycolytic cancer cells that rapidly convert glucose to lactate (13). Lactate oxidation to pyruvate by LDHs further promotes lysosomal acidification and autophagy (14).

LDHs are key enzymes at the core of this adaptive metabolism as they catalyze the terminal reaction of lactate biosynthesis with the interconversion of pyruvate and NADH to lactate and NAD^+^. LDHs function as obligate tetramers constituted by the homoassociation or heteroassociation of two isoenzymes, LDH-H (lactate dehydrogenase heart isozyme; encoded by the *LDHB* gene) and LDH-M (lactate dehydrogenase muscle isozyme; encoded by the *LDHA* gene) (15). These two isoenzymes show very high homology and identity (16). The two LDH homotetramers, lactate dehydrogenase heart isozyme homotetramer (LDH-1) (LDH-H_4_) and lactate dehydrogenase muscle isozyme homotetramer (LDH-5) (LDH-M_4_), are the most extensively studied forms of LDH and constitute appealing targets for cancer therapy (17, 18).

Intense efforts were initially devoted to selective LDH-5 inhibition because of its broad implication in cancer pathogenesis (19, 20). However, more recent reports about the implications of LDH-1 in cancer pathogenesis shed light on LDH-1 inhibition. First, LDH-1 was reported to interact with lysosomal vesicular ATPase, thus regulating autophagy, and is essential for metabolic reprogramming through p53 and Ras mutations (14, 21). Second, the *LDHB* gene was identified to be essential for triple-negative breast cancer (22). Finally, it has been shown that one LDH isoenzyme can compensate for the genetic disruption of the other in order to sustain the Warburg phenotype (23). Altogether, these studies support the idea that dual LDH inhibitors could bring an additional therapeutic value over selective isoenzyme inhibition.

The therapeutic interest for LDH inhibition prompted the development of potent, dual or selective, active-site LDH inhibitors (24, 25, 26, 27, 28, 29, 30). However, despite intense efforts, pharmacological LDH inhibition struggled to translate to *in vivo* activity (27, 29, 31, 32). In fact, LDHs are usually recognized as poorly druggable targets, and different reasons can account for this. First, LDH active-site inhibitors face a challenge in achieving selectivity over other dehydrogenases, notably because of a common NAD-binding domain. For instance, gossypol derivatives, which are among the first LDH inhibitors reported (20), demonstrated significant inhibition toward other dehydrogenases (33). Second, the LDH catalytic site presents nonoptimal physicochemical properties with high solvent exposure and hydrophilicity, leading to challenging absorption, distribution, metabolization, and excretion properties for most LDH active-site inhibitors (27, 29, 34). Finally, an inherent difficulty in achieving therapeutic LDH inhibition stems from its high intracellular concentration; LDHs are indeed highly concentrated in cancer cells, with protein concentrations reported in the micromolar range (29). This high cellular concentration often hampers the observation of cell-based inhibition below that micromolar threshold, even for the more potent nanomolar inhibitors reaching micromolar concentrations in tumors (29, 32).

These different challenges to LDH inhibition called for developing new strategies to target this enzymatic family of high therapeutic potential. To this end, tool compounds able to target the LDH oligomeric interface instead of its active site have been recently developed (16, 35, 36, 37). Targeting a protein oligomeric state is still an underexplored strategy that can provide several benefits over active-site targeting and could thus overcome the existing difficulties encountered with LDH orthosteric inhibitors. Targeting LDH self-assembly could indeed lead to the identification of new and potentially more druggable allosteric sites (16). Noteworthy, as LDH subunits can form homotetramers and heterotetramers, the tetrameric interface is shared between the two different isoenzymes. Targeting LDH tetrameric interface can thus yield to molecules disrupting both LDH-1 and LDH-5, which is in line with the current pan-LDH inhibition strategy (16). Moreover, disruptors of protein self-assembly can induce protein misfolding and degradation (38, 39). Therefore, targeting the LDH oligomeric state could reduce its intracellular concentration, leading to substoichiometric inhibition, hence higher efficacy.

To this end, we previously developed and characterized a dimeric model of LDH-H by truncating its N-terminal tetramerization domain (LDH-H truncated [LDH-Htr]) (16). This model allows to study the LDH tetrameric interface and previously led to the identification of a first allosteric site involving the N-terminal residues. The present study reports on the existence of a second allosteric site, based on which we developed a new family of peptidic ligands functioning as LDH destabilizers.

## Results and discussion

### *In silico* mapping of the LDH-1 tetrameric interface identifies a new cluster of interactions

**Figure 1:**
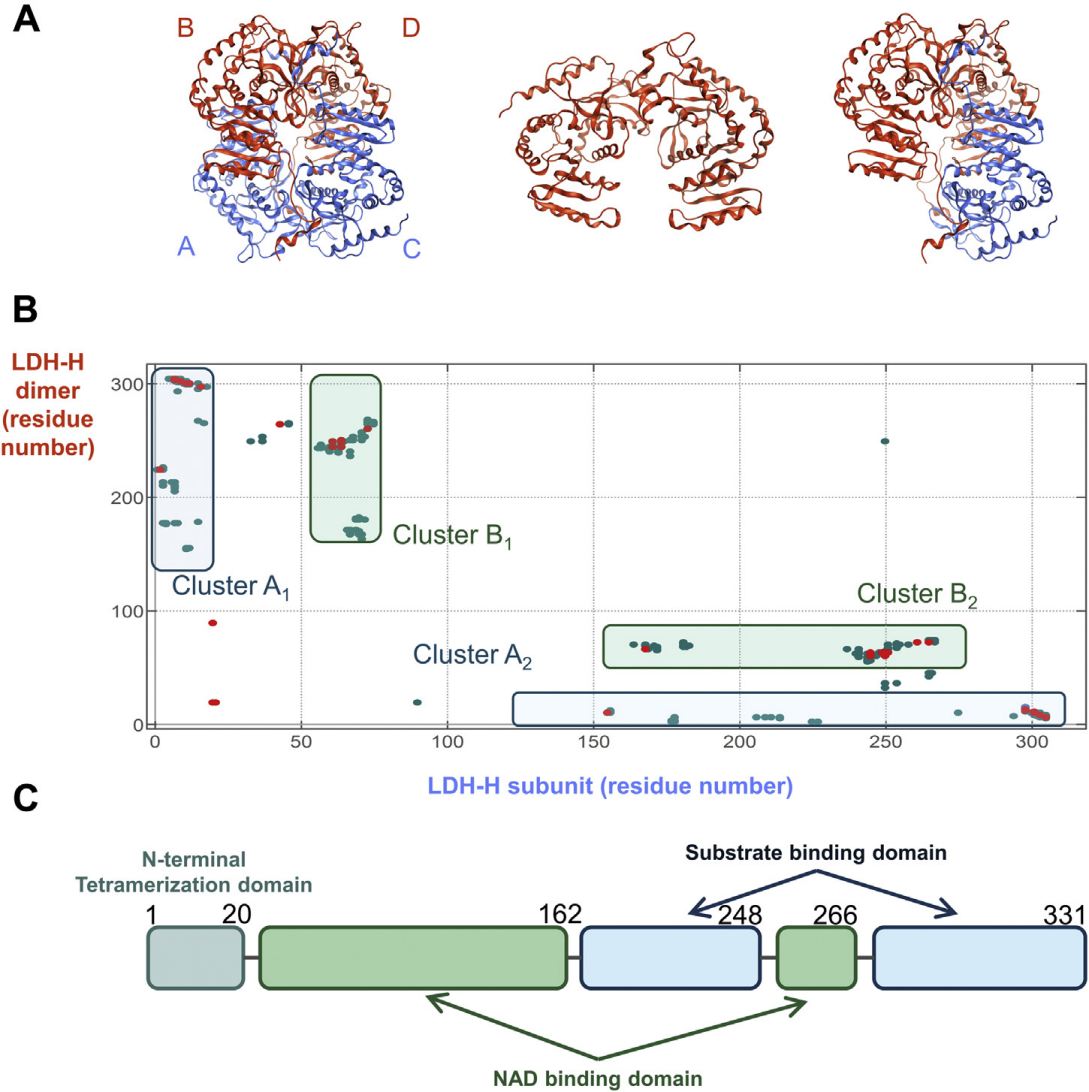
**Interaction mapping of LDH-H tetrameric interface highlights two main clusters.***A*, *Left*, X-ray crystallographic structure of LDH-1 (LDH-H_4_) as a dimer of dimers with the two dimers colored differently (subunits A and C as well as B and D). *Middle*, model of dimeric LDH-Htr. *Right*, model of dimeric LDH-H interacting with a single LDH-H subunit used to highlight LDH tetrameric interface (Protein Data Bank ID: 1I0Z). *B*, mapping of the interaction between an LDH-H subunit (C) and LDH-1 tetrameric interface (dimer B–D) using the Molecular Operating Environment software. The *x*-axis and *y*-axis represent the residue numbers of dimers B to D and subunit C, respectively. This mapping identifies two clusters of interaction, clusters A and B. *C*, representation of the different domains of native LDH-1 (UniProt: P07195). Residue numbers are scaled to *x*-axis (*B*). LDH-1, lactate dehydrogenase heart isozyme homotetramer; LDH-H, lactate dehydrogenase heart isozyme; LDH-Htr, lactate dehydrogenase heart isozyme truncated.

LDH quaternary state is a “dimer of dimers” (Fig. 1*A*) (40, 41). According to X-ray structures, three different subunit orientations could account for LDH dimeric conformation. We have indeed previously found that LDH N-terminal domain truncation leads to dimers (LDH-Htr) (Fig. 1*A*) (16). In agreement with previous studies (35), only the association of dimers A to C and B to D in a tetramer can explain the role of this N-terminal domain in the stabilization of the tetrameric state (Fig. 1*A*). Based on this hypothesis, we first mapped the interactions made by one subunit with an LDH dimer (A–C or B–D) using the Molecular Operating Environment (MOE; Chemical Computing Group [ChemComp]) software (42). Mapping these contact points highlighted two clusters: A (A_1_ and A_2_) and B (B_1_ and B_2_) (Fig. 1*B*). Clusters A_1_ and A_2_ corresponded to the LDH N-terminal tetramerization domain (Fig. 1*C*) and to its related tetramerization site, respectively, which were previously investigated (16). Clusters B_1_ and B_2_ matched with a 22-amino acid α-helix and its interacting site, which to our knowledge were never reported before. Interestingly, the sequence corresponding to cluster B_1_ was highly conserved among vertebrates (Fig. S1). Overall, clusters A_1_ and B_1_ corresponded to continuous epitopes interacting with discontinuous oligomerization sites A_2_ and B_2_.

### LDH-Htr behaves as a weak tetramer through cluster B

**Figure 2:**
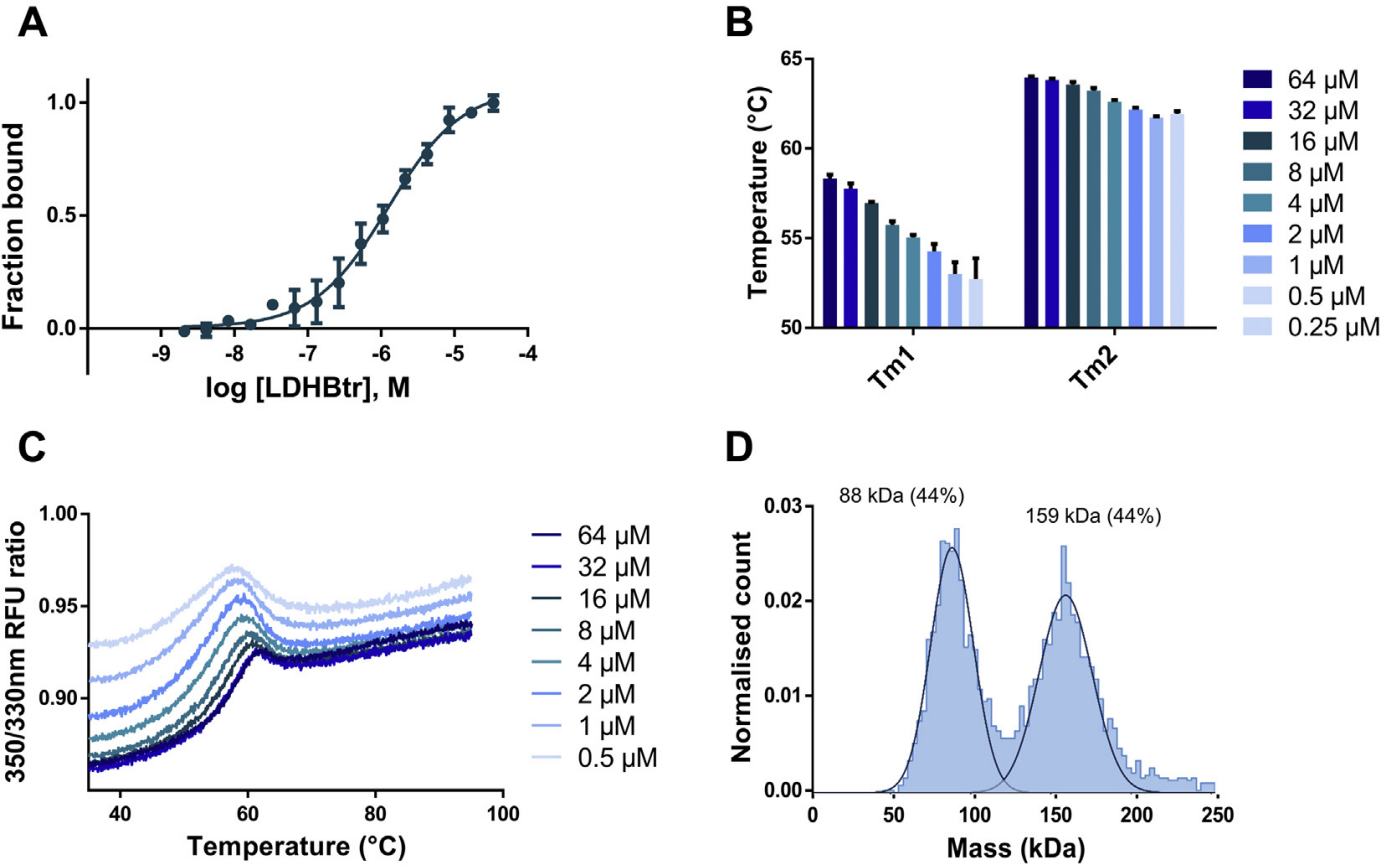
**LDH-Htr behaves as a weak tetramer.***A*, evaluation of LDH-Htr self-interaction using microscale thermophoresis at a 20 s “on time” (n = 3) (*K*_*d*_ = 1.25 μM [0.96–1.62 μM]). *B*, evaluation by nanoscale differential scanning fluorimetry (nanoDSF) of the impact of LDH-Htr melting temperature depending on its subunit concentration (n = 3). *T*_m1_ and *T*_m2_ refer to the two transitions observed for LDH-Htr denaturation pattern. *C*, nanoDSF profile of LDH-Htr at various concentrations (n = 3). RFU, relative fluorescent unit. *D*, mass photometry of LDH-Htr with the calculated molecular weights of the complex in solution and their relative intensity indicated above the peaks (theoretical molecular weight of the dimer is 73.2 kDa). LDH-Htr, lactate dehydrogenase heart isozyme truncated.

Next, we aimed to confirm this interaction model and the anticipated symmetry axis of LDH dimers using our model of dimeric LDH (LDH-Htr) (16). According to the interaction map, LDH-Htr lacks cluster A_1_ but still possesses clusters A_2_, B_1_, and B_2_. We thus reasoned that LDH-Htr might still be able to self-interact at high concentrations *via* cluster B. Comparison between LDH-Htr and LDH-1 elution profiles by size-exclusion chromatography indeed suggested that LDH-Htr could be in an equilibrium between tetramers and dimers (16). Consistently, the evaluation of LDH-Htr self-interaction by microscale thermophoresis (MST) revealed that the dimeric protein interacts with itself at high concentrations (Fig. 2*A*; *K*_*d*_ = 1.25 μM [0.96–1.62 μM]). According to our model, this interaction can only be the result of LDH-Htr forming dimers of dimers through cluster B. Monitoring this interaction using MST hence provided valuable information on the overall potency of cluster B. We then evaluated whether LDH-Htr self-association could stabilize the protein complex, as the oligomerization of a protein often results in its stabilization (43). LDH-Htr denaturation profile was evaluated using nanoscale differential scanning fluorimetry (nanoDSF) and revealed that the protein exhibited a concentration-dependent destabilization (Fig. 2*B*) and conformational change (Fig. 2*C*). We also evaluated LDH-Htr oligomeric state using mass photometry (MP). MP is a recent technique that allows for single-molecule detection and mass measurement in solution based on light scattering (44). MP analysis of LDH-Htr revealed an equilibrium between dimers and tetramers in solution (Fig. 2*D*). Altogether, these results demonstrated that the truncation of the LDH N-terminal domain does not entirely prevent the protein from forming tetramers. This ability of the truncated dimers to interact and form weak tetramers validated our *in silico* model and provided valuable information about this new tetrameric interface.

### Identification of peptide ligands of the LDH tetrameric interface

**Figure 3:**
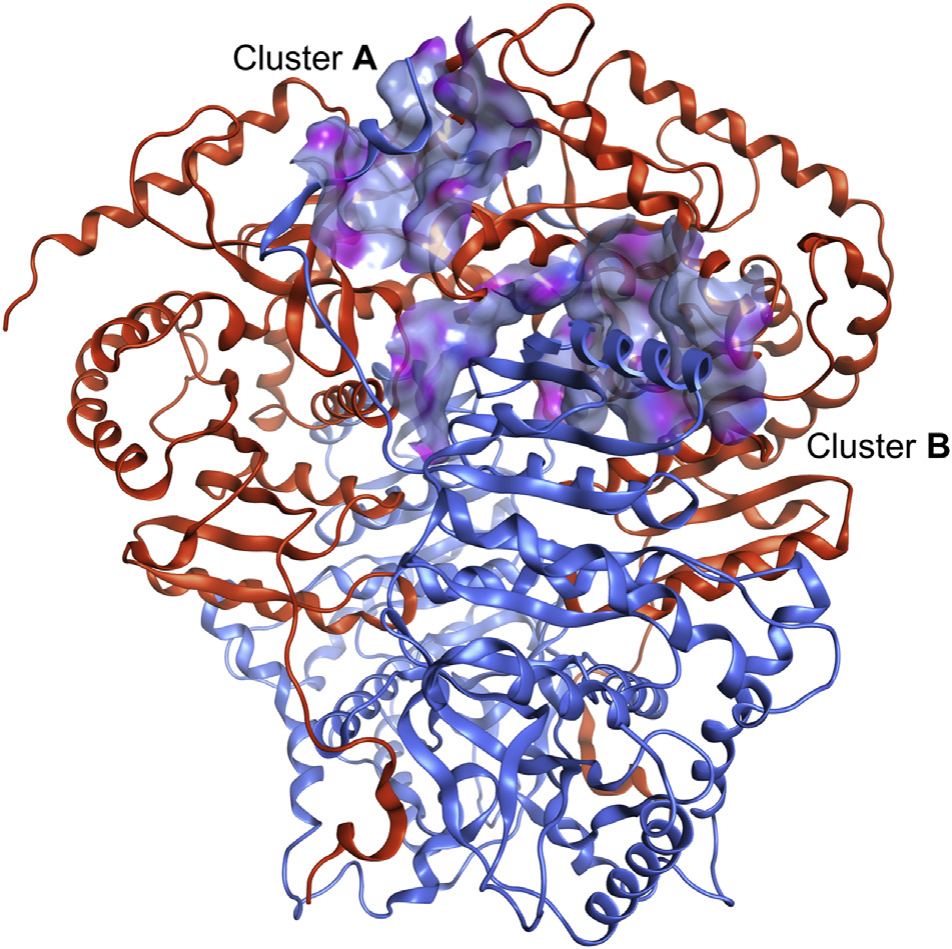
**Structural representation of LDH tetrameric interface shows that cluster B**_**1**_**continuous epitope is a long α-helix.** Representation of clusters A_1_ and B_1_ (*blue*, *ribbon*) interacting with clusters A_2_ and B_2_ (*orange*, *surface*). The surface corresponds to the molecular surface of LDH-H clusters A_2_ and B_2_ colored by lipophilicity (*gray blue*: lipophilic; *pink*: hydrophilic). This representation was generated from the LDH-1 crystallographic structure and further minimized using Molecular Operating Environment software (Protein Data Bank ID: 1I0Z). LDH, lactate dehydrogenase; LDH-1, lactate dehydrogenase heart isozyme homotetramer; LDH-H, lactate dehydrogenase heart isozyme.

We then set out to further characterize the continuous epitope B_1_ in order to identify peptides targeting the LDH tetrameric interface. As discussed previously, cluster B_1_ corresponds to a 22-amino acid peptide folding into a long and “kinked” α-helix ended by a short loop (Fig. 3). We thus decided to study the interaction between “cluster B_1_”-derived peptide (named LP-22, LEDKLKGEMMDLQHGSLFLQTP) and the LDH-H tetrameric interface. To that end, we performed a set of biophysical evaluation using NMR water–ligand observed *via* gradient spectroscopy (WaterLOGSY), MST, and nanoDSF experiments.

**Figure 4:**
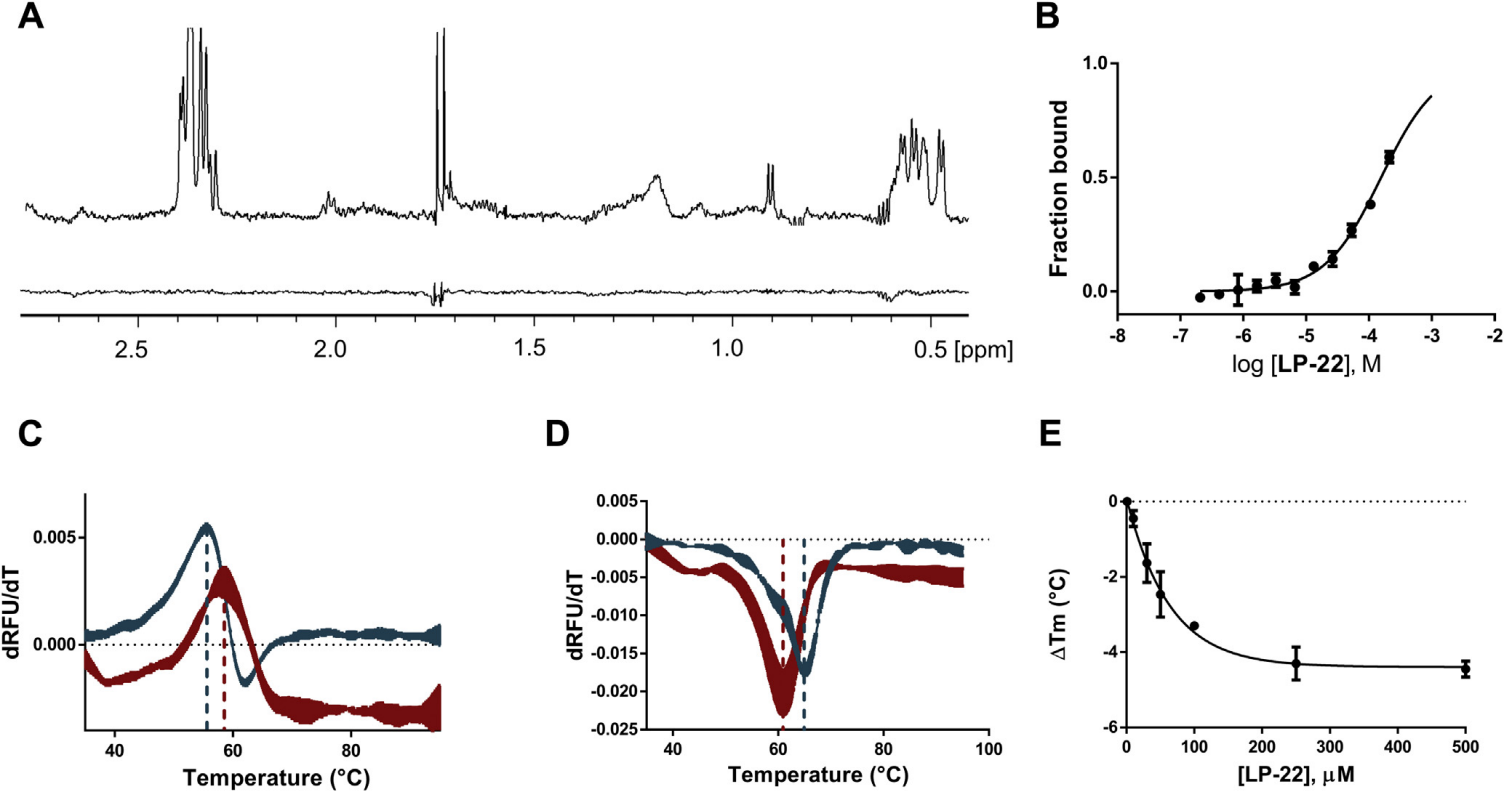
**Peptide LP-22 interacts at the LDH tetrameric interface, destabilizes tetrameric LDH, and stabilizes dimeric LDH.***A*, WaterLOGSY spectra of the interaction of LP-22 (400 μM) with dimeric LDH-Htr (*up*) and tetrameric LDH-1 (*down*) at 15 μM. *B*, MST binding curves between LP-22 and LDH-Htr. Binding curves were extracted from the MST traces at a 1.5 s MST on time (n = 3). *C*, nanoDSF denaturation of dimeric LDH-Htr (15 μM) with (*red*) and without (*teal*) LP-22 (500 μM) (Δ*T*_m_ = 2.8 °C; n = 3). *D*, nanoDSF denaturation of tetrameric LDH-5 (300 nM) with (*red*) and without (*teal*) LP-22 (250 μM) (Δ*T*_m_ = −4.3 °C; n = 3). *E*, Δ*T*_m_ (°C) of tetrameric LDH-5 (300 nM) as a function of LP-22 concentration (EC_50_ = 47 μM [32–68 μM]; n = 3). LDH, lactate dehydrogenase; LDH-1, lactate dehydrogenase heart isozyme homotetramer; LDH-5, lactate dehydrogenase muscle isozyme homotetramer; LDH-Htr, LDH-H truncated; LP-22, cluster B1-derived peptide; MST, microscale thermophoresis; nanoDSF, nanoscale differential scanning fluorimetry; RFU, relative fluorescent unit; WaterLOGSY, water–ligand observed *via* gradient spectroscopy.

Strikingly, WaterLOGSY experiments showed that LP-22 undergoes a saturation transfer with dimeric LDH-Htr, but not with the tetrameric LDH-1, thus demonstrating that it interacts at the LDH tetrameric interface (Fig. 4*A*). MST further validated this interaction with an estimated *K*_*d*_ of 156 μM with LDH-Htr (Fig. 4*B*). Thermal shift experiments using nanoDSF revealed a stabilization (Δ*T*_m_ = 2.8 °C at 500 μM) (Fig. 4*D*) of dimeric truncated LDH-Htr with LP-22, consistent with an interaction occurring at the exposed oligomeric interface and with the stabilization that usually follows a ligand–protein interaction because of an increase in its Gibbs free energy of unfolding (45). On the opposite, LP-22 destabilized tetrameric LDH in a concentration-dependent manner (Fig. 4, *E* and *F*) with an EC_50_ = 47 μM (32–68 μM). These results are coherent with the observation that ligands interacting at oligomeric interfaces often induce protein thermal destabilization (46). The LDH-5 tetramer was more destabilized than LDH-1, consistent with a difference in the stability of these two protein complexes that we previously reported (Fig. S2) (16). Interestingly, a recent report of selective LDH-5 inhibitors disclosed an inhibitor interacting at LDH-5 tetrameric interface near cluster B. This inhibitor was highly selective toward LDH-5, which is coherent with the lower stability of LDH-5 tetrameric complex (36). Such selectivity profile is also consistent with the higher destabilizing effect that LP-22 displays on LDH-5 compared with LDH-1. Consistently with our previous report on LDH tetramer disruptors (16), LDH destabilization was also dependent on protein concentration, as an increasing amount of subunit reverted the effect (Fig. S2). Overall, these results consistently demonstrated that LP-22 interacts at the LDH tetrameric interface and destabilizes the tetrameric enzyme.

### Biophysical and computational experiments identify LP-22 essential binding region

**Figure 5:**
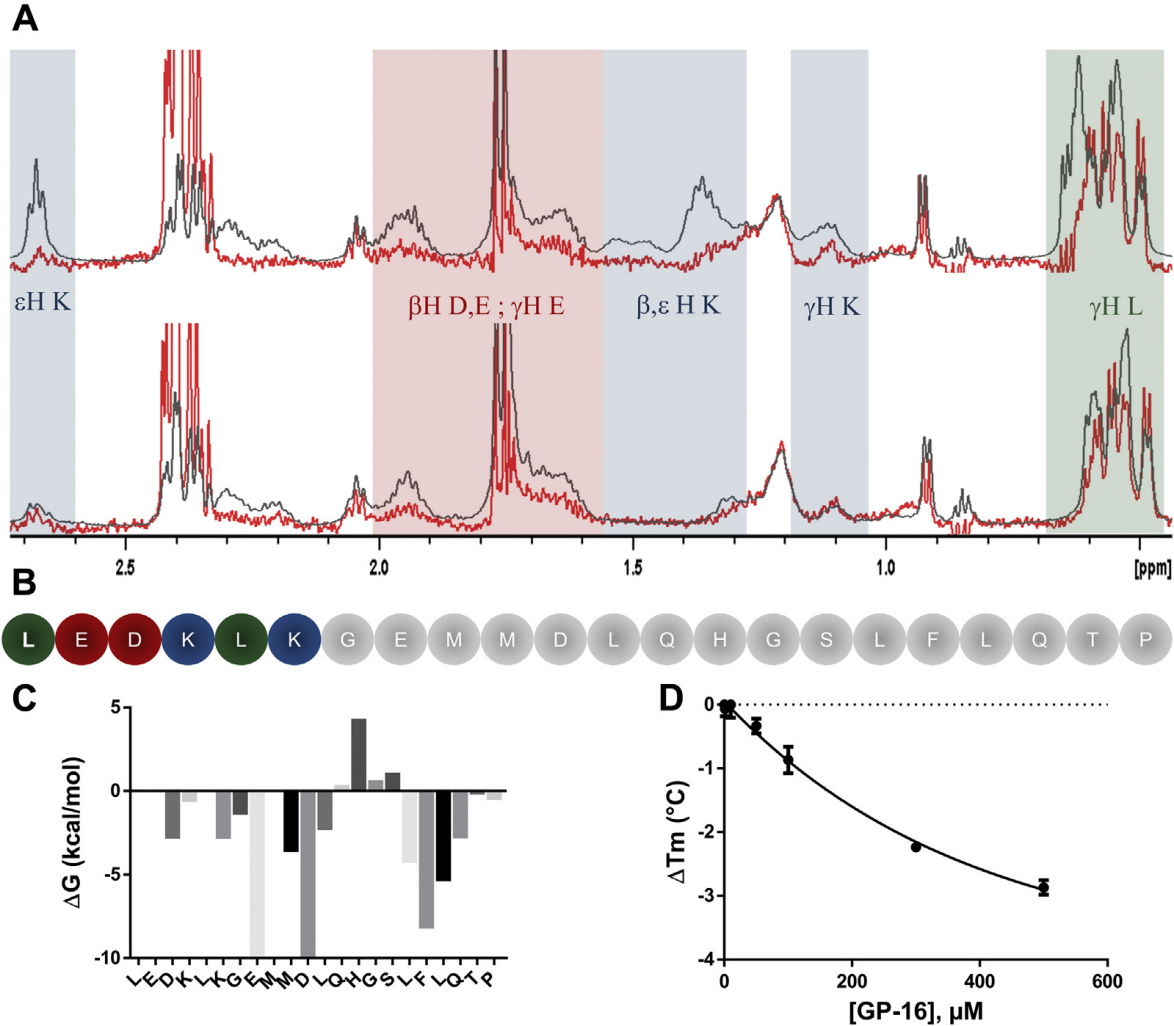
**LP-22 N-terminal trimming leads to GP-16 with a similar interaction profile.***A*, comparison of the difference between LP-22 (*up*) and GP-16 (*bottom*) WaterLOGSY (*red*) and 1H (*black*) NMR spectra in the presence of 15 μM of LDH-Htr. Signals that appear in the 1H spectra but not in WaterLOGSY correspond to noninteracting residues. LP-22 spectra highlight that some lysine (*blue*), glutamate (*red*), aspartate (*red*), and leucine (*green*) residues do not interact with LDH-Htr. These noninteracting signals are no longer present on the GP-16 spectra (*down*). *B*, peptide sequence of LP-22. Colored residues correspond to the residues that do not interact according to ΔG calculation and WaterLOGSY analysis. *C*, calculation of LP-22 residue contribution to the overall free energy of binding using the Molecular Operating Environment software. *D*, differences in melting temperature (Δ*T*_m_, °C) of tetrameric LDH-5 (300 nM) as a function of GP-16 concentration (EC_50_ = 262 μM [142–383 μM]; n = 3). LDH-5, lactate dehydrogenase muscle isozyme homotetramer; LDH-Htr, LDH-H truncated; LP-22, cluster B1-derived peptide; WaterLOGSY, water–ligand observed *via* gradient spectroscopy.

We next compared LP-22 WaterLOGSY and ^1^H-NMR spectra. Because WaterLOGSY is a ligand-based NMR spectroscopy that relies on protein–ligand saturation transfer, LP-22 residues that do not interact with the protein will be absent of the WaterLOGSY spectrum (47). A careful comparison between LP-22 ^1^H and WaterLOGSY spectra highlighted ^1^H chemical shift regions characteristic to lysine, glutamate, aspartate, and leucine aliphatic regions that were not undergoing saturation transfer (Fig. 5, *A* and *B*). Calculation of the contribution of each residue to the peptide-binding free energy further suggested that N-terminal LP-22 residues LEDKLK, a region rich in those particular amino acids, did not account for much of LP-22 binding energy (Fig. 5*C*). Removal of these six N-terminal residues led to GP-16 (GEMMDLQHGSLFLQTP), a peptide that had a similar WaterLOGSY spectrum (Fig. 5*A*), thus suggesting that the two peptides were interacting very similarly. MST indicated that the interaction with dimeric LDH was slightly weakened, with a *K*_*d*_ = 240 μM (Fig. S3). Consistently, nanoDSF established that GP-16 could still destabilize tetrameric LDH in a concentration-dependent manner (EC_50_ = 262 μM [142–383 μM]) (Fig. 5*D*).

### Probing GP-16 and LDH-H tetrameric interface hot spots

**Figure 6:**
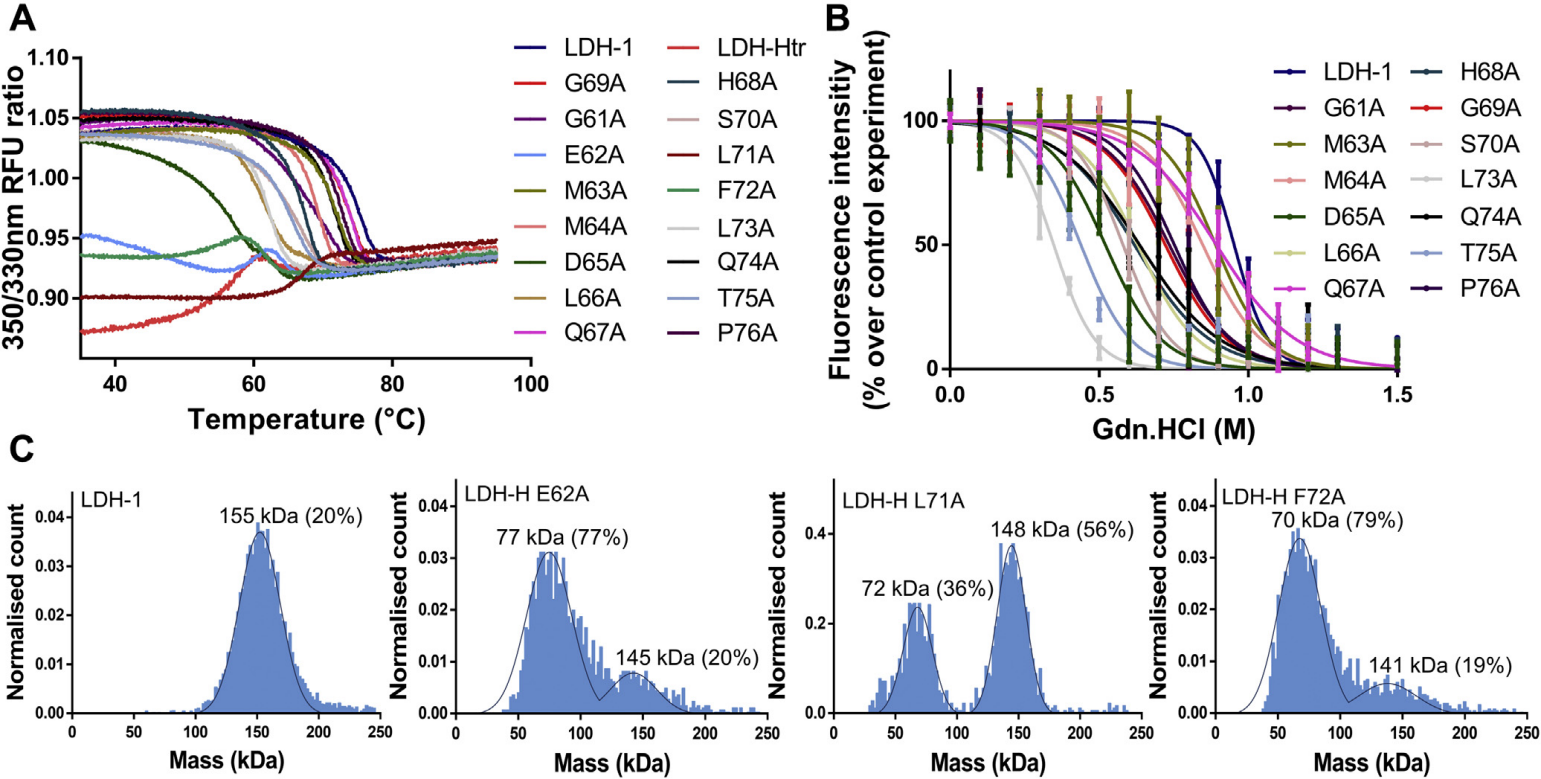
**Mutations of cluster B**_**1**_**unravel key residues for LDH tetramerization.***A*, screening of LDH-H variants using nanoDSF. Changes in the 350/330 nm fluorescence emission indicate blue or red shifts and are representative of unfolding events (n = 6). *B*, dissociation of the homotetrameric form of LDH-H and of its variants at 50 μg/ml (1.3 μM) upon addition of guanidinium·hydrochloride. Tryptophan fluorescence intensity was followed at λ_exc_ = 286 nm and λ_em_ = 350 nm as a direct reporter of LDH-1 tetrameric integrity (n = 6) (49). *C*, mass photometry was performed for different LDH-H variants with the experimental molecular weights of the complexes in solution and their relative intensities. Theoretical molecular weight of the tetramer = 155 kDa; theoretical molecular weight of the dimer = 78 kDa. Profiles of the other variants can be found in Fig. S4. λ_em_, wavelength of emission; λ_exc_, wavelength of excitation; LDH, lactate dehydrogenase; LDH-1, lactate dehydrogenase heart isozyme homotetramer; LDH-H, lactate dehydrogenase heart isozyme; nanoDSF, nanoscale differential scanning fluorimetry; RFU, relative fluorescent unit.

Computational and biophysical data suggested that the GP-16 sequence represents an essential binding region of the LDH tetrameric interface. To verify this hypothesis, we probed the contribution of each residue of cluster B_1_ to the stability of LDH-H oligomeric state. To that end, we performed an alanine scanning of the LDH-1 sequence corresponding to GP-16. We thus designed, produced, and purified the 16 corresponding LDH-H recombinant alanine variants and evaluated these mutants for their thermal and chemical stability (Fig. 6, *A* and *B* and Table 1). These variants were also evaluated by MP (Table 1 and Fig. 6*C* and Fig. S4).

**Table 1:** Oligomeric state and stability of LDH-H variants^a^

Protein	Molecular weight (kDa)	EC_50_ (M)	*T*_m_ (°C)	Ratio 350/330 nm
LDH-1	155 ± 17	0.953 ± 0.012	74.5 ± 0.1	1.04
LDH-Htr	88 ± 13	<0.1	57.5 ± 0.1	0.87
G61A	142 ± 10	0.735 ± 0.018	69.8 ± 0.1	1.05
E62A	77 ± 18	<0.1	58.9 ± 0.1	0.95
M63A	141 ± 14	0.891 ± 0.012	71.6 ± 0.1	1.04
M64A	149 ± 18	0.845 ± 0.010	68.7 ± 0.1	1.04
D65A	143 ± 12	0.521 ± 0.012	56.4 ± 0.1	1.03
L66A	137 ± 23	0.630 ± 0.011	61.1 ± 0.1	1.03
Q67A	134 ± 16	0.893 ± 0.010	73.1 ± 0.1	1.04
H68A	154 ± 15	0.619 ± 0.016	67.2 ± 0.2	1.05
G69A	153 ± 18	0.722 ± 0.015	73.5 ± 0.1	1.05
S70A	144 ± 13	0.580 ± 0.009	66.0 ± 0.1	1.04
L71A	148 ± 12	<0.1	67.1 ± 0.1	0.90
F72A	70 ± 17	<0.1	53.9 ± 0.1	0.94
L73A	137 ± 10	0.348 ± 0.008	62.0 ± 0.1	1.03
Q74A	143 ± 10	0.636 ± 0.020	71.3 ± 0.1	1.05
T75A	145 ± 10	0.439 ± 0.012	64.9 ± 0.1	1.04
P76A	142 ± 17	0.752 ± 0.011	72.2 ± 0.1	1.05

a Reported values are means ± SEM for melting temperatures and EC_50_ (n = 6) and means ± SD for the molecular weight (the values presented here were obtained with MP from one measurement and were repeated at least 3 times with similar results). The reported molecular weights correspond to the main oligomeric state of the protein.

**Figure 7:**
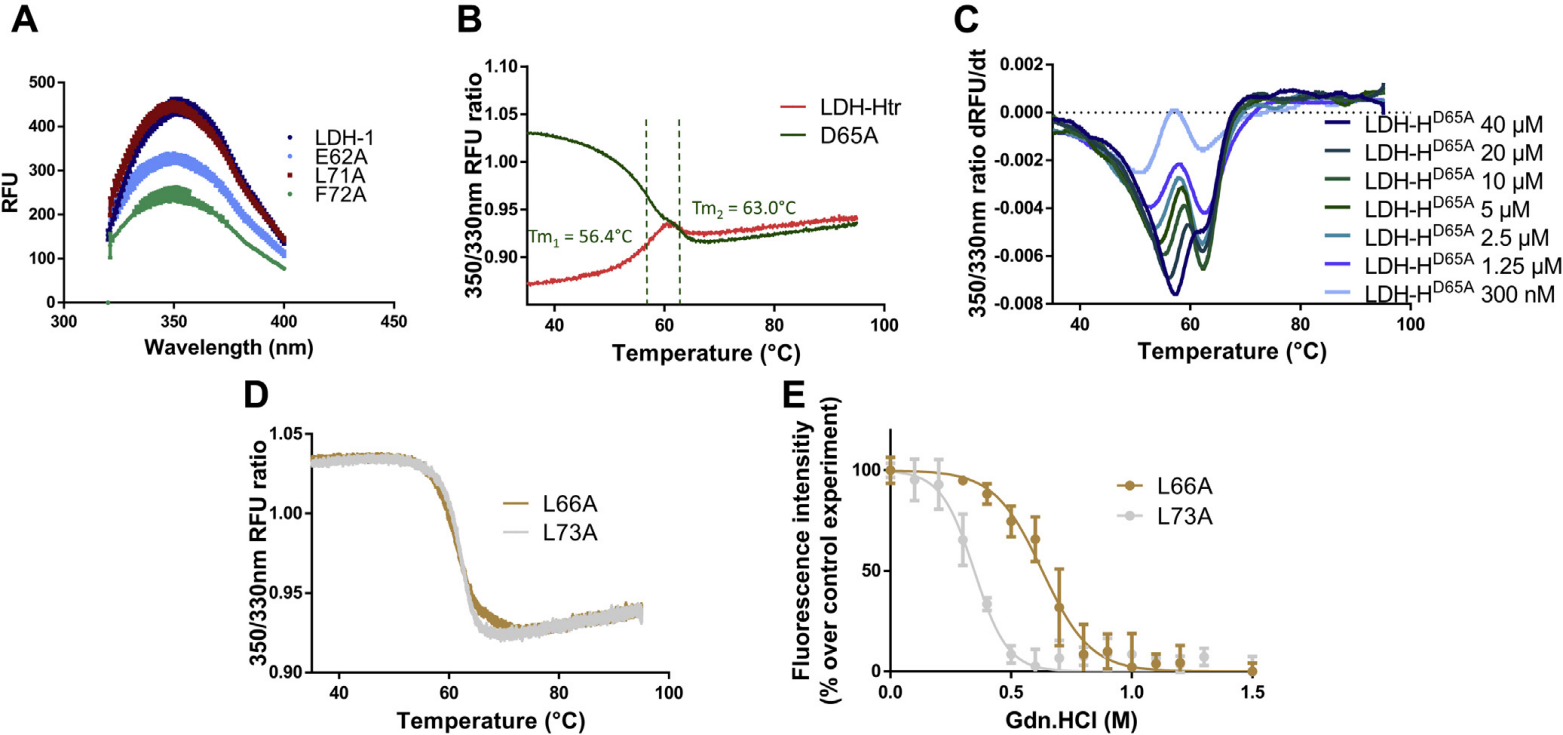
**The exploitation of orthogonal methods highlights the impact of key mutations on LDH-H tetrameric stability.***A*, tryptophan fluorescence spectra of different LDH-H variants (1.3 μM). λ_exc_ = 286 nm (n = 6). *B*, nanoDSF profiles of LDH-H^D65A^ and LDH-Htr (n = 6). *C*, nanoDSF profile of LDH-H^D65A^ at different concentrations. Data are represented as the derivative of the 350/330 nm fluorescence ratio to highlight the apparition of the second unfolding event (n = 3). *D*, nanoDSF profiles of LDH-H^L66A^ and LDH-H^L73A^ (n = 6). *E*, fluorescence intensity of tetrameric LDH-H^L66A^ and LDH-H^L73A^ at 50 μg/ml (1.3 μM) upon addition of guanidinium·hydrochloride (n = 6). λ_exc_, wavelength of excitation; LDH-H, lactate dehydrogenase heart isozyme; LDH-Htr, LDH-H truncated; nanoDSF, nanoscale differential scanning fluorimetry; RFU, relative fluorescent unit.

Among the different single-point alanine mutations, three of them significantly impacted the LDH-1 oligomeric state, with variants E62A and F72A behaving mainly as dimers in solution and variant L71A behaving as a mixture of tetramers and dimers according to MP results (Fig. 6*C*). Consistently, nanoDSF experiments showed that LDH-H^F72A^ and LDH-H^E62A^ exhibited denaturation patterns comparable to dimeric LDH-Htr, with a lower initial 350/330 nm ratio and a red shift instead of the blue shifts usually observed for tetrameric LDH variants (Fig. 6*A*). Interestingly, LDH-H^L71A^ exhibited a lower initial ratio and a red shift as well but a *T*_m_ 10 °C higher than LDH-Htr. This mixed profile is coherent with the mixture of dimers and tetramers that appear to be present in solution with this variant. Comparison of the tryptophan fluorescence spectra of these variants with LDH-1 showed decays in fluorescence intensity characteristic of the dimeric forms of LDHs for variants E62A and F72A but not L71A (Fig. 7*A*) (16).

Mutations of L73 and D65, two other hot spots previously suggested by *in silico* analysis, resulted in tetrameric variants displaying a significant reduction of stability as assessed by both thermal and chemical denaturation methods (Fig. 5 and Table 1). In line with the expected reduction of tetrameric stability, dilution experiments of the D65A variant resulted in concentration-dependent destabilization of the protein and in the apparition of a second unfolding event (Fig. 7, *B* and *C*). Interestingly, variants L66A and L73A showed similar stabilities by nanoDSF, with *T*_m_ of 61.1 and 62.0 °C, respectively. However, chemical denaturation experiments demonstrated a striking difference in stability between these two mutants, with EC_50_ of 0.630 and 0.348 M, respectively (Fig. 7, *D* and *E* and Table 1). MP experiments validated the presence of an equilibrium between dimers and tetramers for L73A but not for L66A (Fig. S4). In accordance with our *in silico* calculation and available crystallographic data (48), these results confirm that mutation L73A reduces LDH-1 oligomeric stability. In contrast, mutation L66A appears to impact the stability of the protein differently, for instance, by disturbing its hydrophobic core. These results further highlight the importance of exploiting orthogonal methods when assessing the impact of a mutation on protein stability. Other mutations resulted in tetrameric proteins displaying a medium to low variation of their chemical and thermal stability compared with wildtype LDH-1, highlighting the lesser importance of these residues for the oligomeric state of the protein (Table 1).

**Figure 8:**
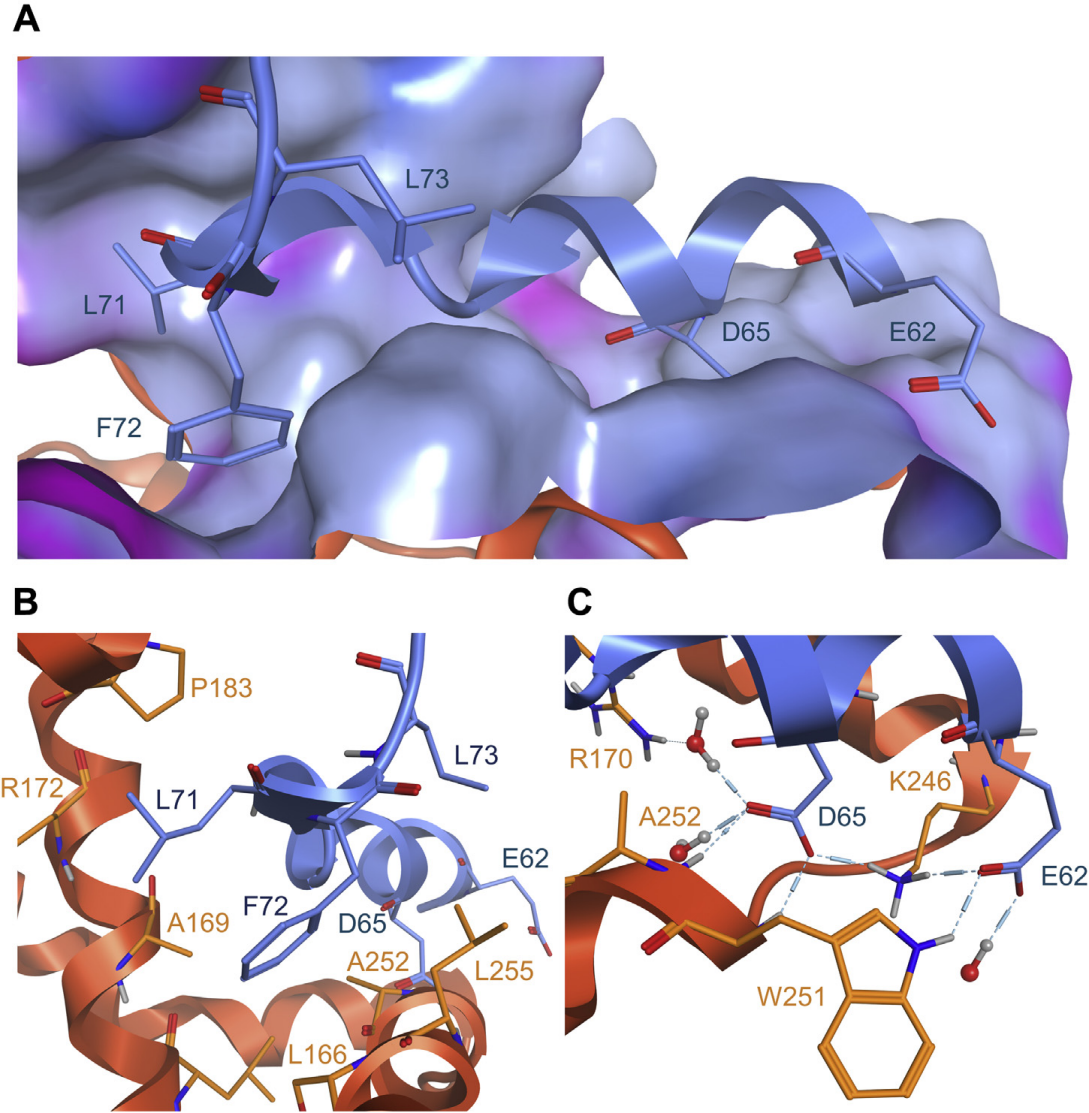
**Structural model of the interaction between cluster B**_**1**_**hot spots and cluster B**_**2**_**.***A*, interaction of the sequence corresponding to peptide GP-16 with cluster B_2_. The surface corresponds to the molecular surface of LDH-H cluster B_2_ colored for lipophilicity (*gray blue*: lipophilic; *pink*: hydrophilic). *B*, focus on the hydrophobic hot spot of cluster B_1_ with the interaction made by L71, F72, and L73 (*blue*) with cluster B_2_ (*orange*). *C*, focus on the hydrophilic hot spot of cluster B_1_ with the interaction made by D65 and E62 (*blue*) with cluster B_2_ (*orange*). This representation was isolated from the LDH-1 crystallographic structure and further minimized using the Molecular Operating Environment software (Protein Data Bank ID: 1I0Z). LDH-1, lactate dehydrogenase heart isozyme homotetramer; LDH-H, lactate dehydrogenase heart isozyme.

Overall, the different stabilities of the mutants coherently matched with our *in silico* prediction of the ΔG of interaction and highlighted new molecular determinants of the LDH tetrameric interface (Fig. 5*C*). Cluster B_1_ hot spots are constituted by the two negatively charged amino acids, E62 and D65, and by the three consecutive hydrophobic residues, L71, F72, and L73. Based on the crystallographic structure (protein data bank ID: 1I0Z), E62 and D65 are involved in a hydrogen bond network with water and neighboring residues R170, K246, A252, and W251. L71, F72, and L73 perform hydrophobic interactions between each other and with residues L166, A169, P183, A252, and L255 (Fig. 8). Interestingly, cluster B_1_ is constituted of both polar and apolar hot spots, which contrast with the purely lipophilic hot spots that we had previously identified in the LDH tetramerization arm (16). Therefore, we anticipate that GP-16–derived peptides represent promising starting points for future optimization toward LDH tetramers disruptors acting at cluster B_2_ tetramerization site.

## Conclusion

Over the past years, intense efforts were devoted to the development of LDH inhibitors (17). Unfortunately, the polarity of LDH active site and high intracellular concentrations of the enzyme have challenged the discovery of LDH inhibitors displaying potent and durable *in vivo* inhibition (29, 34). Recently, new advances in the development of ligands targeting LDH oligomeric interface have offered new avenues toward LDH inhibition (16, 35, 36). Targeting protein self-association is an emerging concept in drug design that can bring several advantages over classical orthosteric inhibition. First, targeting the LDH oligomeric interface could unravel new allosteric sites, potentially leading to compounds displaying improved drug-like features compared with LDH active-site inhibitors. Second, molecules interacting at a protein homomeric interface can lead to its destabilization and degradation (43), providing compounds with a substoichiometric effect. In this study, we report the identification and characterization of a new LDH tetrameric interface and its essential residues, using a combination of MP, nanoDSF, and chemical stability experiments. These results are in accordance with previous computational studies that suggested that this region is involved in LDH tetramerization (35, 40). Furthermore, we report the identification of a family of peptidic ligands that target the tetrameric interface of LDH, destabilize the tetrameric LDH, and stabilize the dimeric LDH-Htr. Altogether, this work provides a structural characterization of the molecular determinant of the LDH tetrameric interface as well as valuable pharmacological tools for the future development of compounds targeting the LDH oligomeric state.

## Experimental procedures

### Chemicals and peptides

All reagents were purchased from different chemical suppliers and used without further purification. Peptides were purchased from Genecust (https://www.genecust.com). Structure conformity and purity grade (>95%) were assessed by analytical HPLC analysis and MS. Peptide GP-16 was amidated and acetylated, respectively, at its C and N termini, and LP-22 was only amidated at his C terminus.

### Production and purification of human LDH proteins

The *hLDHB* nucleotidic sequences used to produce full-length, truncated, and variant LDH-H proteins inserted in a pET-28a expression vector were ordered from Genecust. NdeI and Bpu1102I restriction sites were used for sequence insertion and allowed for an N-terminal 6-His tag addition. Protein production and purification were performed following a previously described procedure (16). Recombinant plasmids were then transformed in host bacterium *Escherichia coli* Rosetta (DE3). Transformants were cultured in terrific broth medium supplemented with 50 μg/ml kanamycin and 34 μg/ml chloramphenicol at 37 °C until an absorbance of 0.8 was reached. LDH expression was induced by the addition of 1 mM IPTG at 20 °C for 20 h. Then, cells were collected by centrifugation at 5000 rpm (rotor 11150; Sigma), 4 °C for 25 min. Pellets were suspended in a lysis buffer (Tris–HCl 50 mM pH 8.5, MgCl_2_ 10 mM, NaCl 300 mM, imidazole 5 mM, and glycerol 10%), and then disrupted by sonication, followed by centrifugation at 4 °C, 10,000 rpm (rotor 12165-H; Sigma) for 30 min. The insoluble fraction was discarded, and 1 μl of β-mercaptoethanol was added per milliliter of soluble fraction. Purification of recombinant proteins was performed using 1 ml His-trap Fast-Flow-crude columns (GE Healthcare) according to the manufacturer's instructions. Finally, protein concentrations were measured using the Bradford method with the Protein Assay Kit (Bio-Rad), and sample homogeneity was assessed by SDS-PAGE with Coomassie brilliant blue as a staining agent.

### NMR

Human LDH-H (full length and truncated)-6-His proteins for 1D NMR were expressed and purified from *E. coli*, as described previously. All experiments were performed on an Ascend Avance III 600 MHz system equipped with a broadband cryoprobe (Bruker) following a previously described procedure (16).

For WaterLOGSY NMR studies, samples were prepared in 10% heavy water containing 50 mM sodium phosphate buffer, pH 7.6, and 100 mM NaCl. The concentration of LDHs was ranging from 15 to 20 μM of monomer. Ligand binding was detected using a WaterLOGSY ephogsygpno.2 avance-version sequence with a 1 s mixing time, 4096 scans were collected at 277 K to yield a 16 K points free induction decay. Water signal suppression was achieved using an excitation-sculpting scheme, and a 50 ms spinlock was used to suppress protein background signals.

### *In silico* evaluation

Calculation of the free binding energy and mapping of the interaction at LDH interface was performed using the MOE software with the LDH-1 crystallographic structure (Protein Data Bank entry: 1I0Z) (48). Following a previously described procedure, tetrameric LDH-1 was generated from the Protein Data Bank crystallographic structure using the MOE bioassembly tool (16). The truncated dimeric version of LDH-1 (LDH-Htr) was generated from the tetrameric complex by removing LDH-H subunits B and D as well as the N-terminal domain of subunits A and C. Minimization was performed before free energy calculation and interaction mapping.

### NanoDSF experiments

NanoDSF was performed following previously described procedures (16).

#### Variant evaluation

Solutions of proteins (LDH-H, LDH-Htr or variants), stored in a 50 mM sodium phosphate, 100 mM NaCl, and 20% glycerol, pH 7.6, were evaluated on a Tycho NT.6 device (NanoTemper Technologies) using concentrations ranging from 25 to 65 μM. According to standard manufacturer's procedures, samples were poured into capillaries and heated up to 95 °C in 3 min, while following fluorescence emission at 330 and 350 nm. Melting temperatures were extracted from the derivative of the 350/330 nm fluorescence ratios upon increasing temperature.

#### Peptide evaluation

Solutions of proteins (LDH-1, LDH-5, or LDH-Htr) with peptides were evaluated on a Tycho NT.6 device. Evaluations were performed in a 50 mM sodium phosphate and 100 mM NaCl (pH 7.6) buffer. According to standard manufacturer's procedures, samples were poured into capillaries and heated up to 95 °C in 3 min, while following fluorescence emission at 330 and 350 nm. Melting temperatures were extracted from the derivative of the 350/330 nm fluorescence ratios upon increasing temperature.

### MST

MST measurements were performed on a NanoTemper Monolith NT.115 instrument (NanoTemper Technologies) using red dye–N-Hydroxysuccinimide fluorescent labeling. LDH-Htr purified to homogeneity was labeled with the Monolith red dye–N-Hydroxysuccinimide second-generation labeling dye (NanoTemper Technologies), according to the supplied protocol. Measurements were performed in 50 mM sodium phosphate, pH 7.6, and 100 mM NaCl containing 0.01% Tween-20 in standard-treated capillaries (NanoTemper Technologies). The final concentration of proteins in the assay was 100 nM. Ligands were titrated in 1:1 dilutions following manufacturer's recommendations. Experiments were performed in triplicates using 40% light-emitting diode power, high MST power, laser on time 20 s and laser off time 3 s. Peptides were evaluated for their thermophoretic pattern, and *K*_*d*_s were extracted from raw data at a 1.5 s MST on time following the manufacturer's instructions.

### MP

Protein landing was recorded using a Refeyn OneMP (Refeyn Ltd) MP system by adding 1 μl of the protein stock solution (1 μM) directly into a 16 μl drop of filtered PBS solution. Movies were acquired for 60 s (6.000 frames) with the AcquireMP (version 2.1.1; Refeyn Ltd) software using standard settings. Data were analyzed using default settings on DiscoverMP (version 2.1.1; Refeyn Ltd) (44). Contrast-to-mass calibration was performed prior to the experiments using a mix of proteins with molecular weights of 66, 146, 480, and 1048 kDa.

### Spectrophotometric experiments

All spectrophotometric experiments were performed with opaque 96-well plates using a Spectramax m2e spectrophotometer (Molecular Devices) following previously described procedures (16).

#### Intrinsic fluorescence assays

Full tryptophan fluorescence spectra were recorded using an excitation wavelength of 286 nm and recording the emission spectra from 320 to 400 nm at room temperature. The raw fluorescence of each experiment was subtracted to a corresponding control experiment without the protein. Experiments were performed in a 50 mM sodium phosphate and 100 mM NaCl, pH 7.6, buffer. For dissociation in subunits, increasing amounts of guanidinium hydrochloride ranging from 0.3 to 2 M were put in contact with the studied proteins (1.3 μM), and fluorescence spectra were recorded afterward.

### Statistics

All quantitative data are expressed as means ± SEM. Error bars were sometimes smaller than symbols. n refers to the total number of replicates. Data were analyzed using the GraphPad 7.0 software (Prism).

## Data availability

All data are contained within the article and the supporting information.

## Supporting information

This article contains supporting information.

## Conflict of interest

The authors declare the following competing financial interest(s): L. T., L. B., P. S., and R. F. are inventors of European Patent Application EP19172347.7, LDH inhibitor polypeptides for use in treatment of cancer; L. T., M. L., P. S., and R. F. are inventors of European Patent Application EP21154636.1, polypeptide inhibitors of LDH activity for use in cancer therapy. The authors declare no other conflicts of interest.
