# Supplementary material for: Discovery of a novel lactate dehydrogenase tetramerization domain using epitope mapping and peptides
Source: J Biol Chem. 2021 Feb 17;296:100422. doi: 10.1016/j.jbc.2021.100422 (PMC8010463; doi:10.1016/j.jbc.2021.100422)
Supplement: Supplemental Figures S1–S4 [file mmc1.docx]

**Discovery of a novel lactate dehydrogenase tetramerization domain using epitope mapping and peptides**

Léopold Thabault,^1,2^ Maxime Liberelle,^1^ Katarina Koruza,^3,4,5^ Esra Yildiz,^1^ Nicolas Joudiou,^6^ Joris Messens,^3,4,5^ Lucie Brisson,^7^ Johan Wouters,^8^ Pierre Sonveaux,^*,$,2^ Raphaël Frédérick^*,$,1^

^1^ Louvain Drug Research Institute (LDRI), Université catholique de Louvain (UCLouvain), B-1200 Brussels, Belgium.

^2^ Pole of Pharmacology and Therapeutics, Institut de Recherche Expérimentale et Clinique (IREC), Université catholique de Louvain (UCLouvain),B-1200 Brussels, Belgium.

^3^ VIB-VUB Center for Structural Biology, B-1050 Brussels, Belgium.

^4^ Brussels Center for Redox Biology, B-1050 Brussels, Belgium.

^5^ Structural Biology Brussels, Vrije Universiteit Brussel, B-1050 Brussels, Belgium.

^6^ Nuclear and Electron Spin Technologies, Louvain Drug Research Institute (LDRI), Université catholique de Louvain (UCLouvain), B-1200 Brussels, Belgium.

^7^ Inserm UMR1069, Nutrition, Growth and Cancer, University of Tours, Tours, France.

^8^ NARILIS, Department of Chemistry, UNamur, University of Namur, B-5000 Namur, Belgium.

^$^ R.F. and P.S. are co-senior authors.

*Corresponding authors: Pierre Sonveaux − Pole of Pharmacology and Therapeutics, Institut de Recherche Expérimentale et Clinique (IREC), Université Catholique de Louvain, B-1200 Brussels, Belgium; Email: pierre.sonveaux@uclouvain.be

Raphaël Frédérick − Louvain Drug Research Institute (LDRI), Université Catholique de Louvain, B-1200 Brussels, Belgium; Email: raphael.frederick@uclouvain.be

**Running title:** Discovery of a lactate dehydrogenase tetramerization domain

**Key-words:** Cancer; Peptides; Biophysics; Oligomerization; Protein-protein interaction; Nuclear magnetic resonance (NMR); Mass photometry; MicroScale Thermophoresis; NanoDSF; Lactate dehydrogenases; Disruptors.

Table of Contents

| Figures | 2 |
| --- | --- |
|  |  |


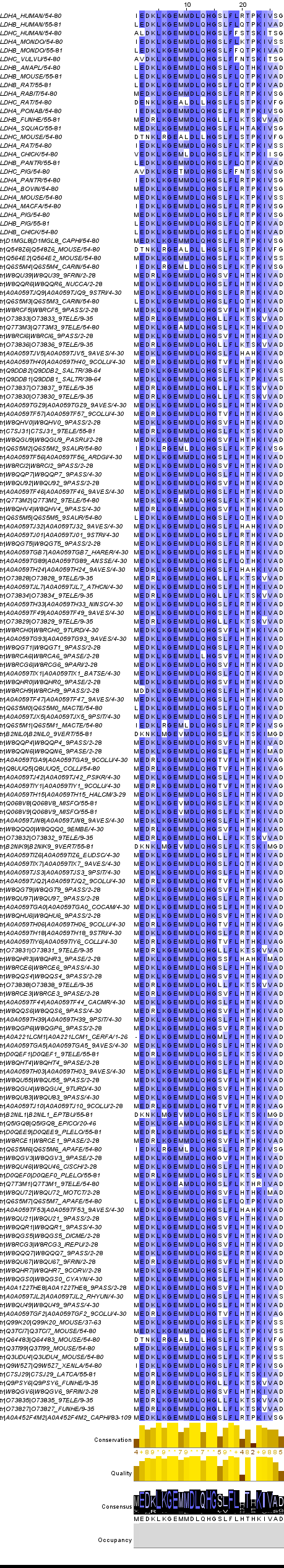
²
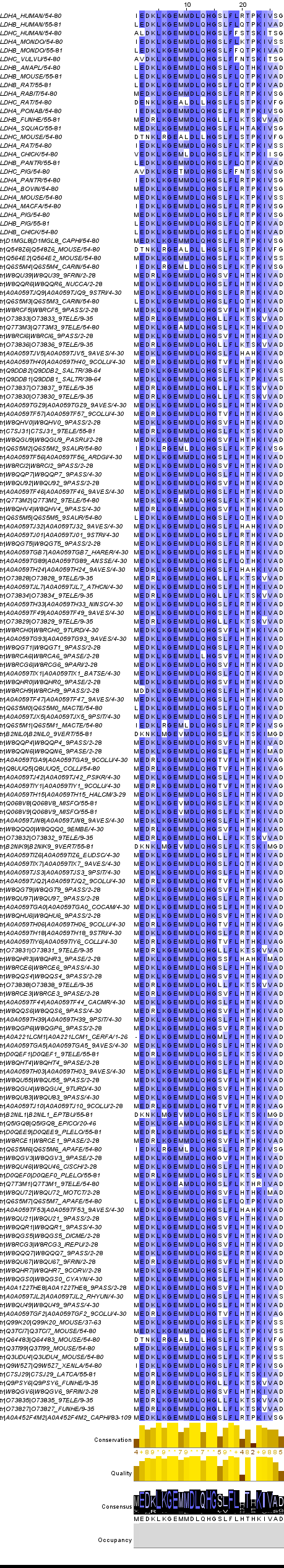

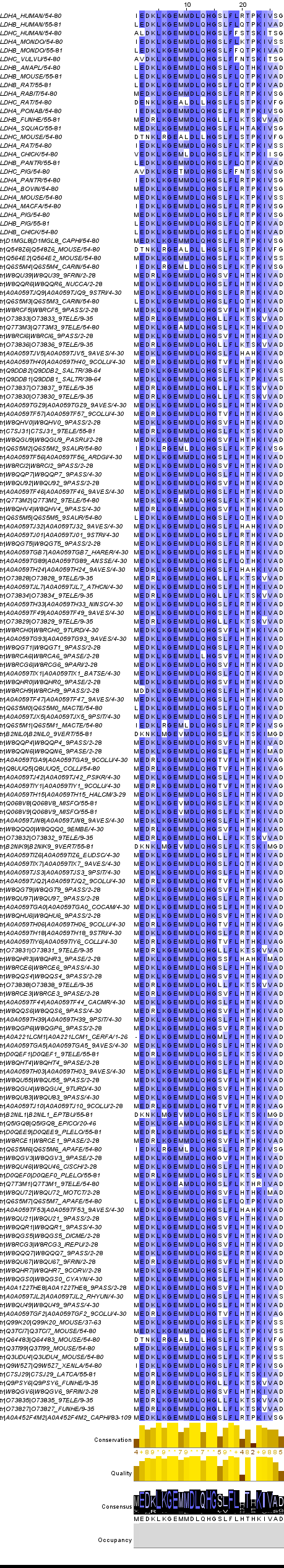


**Figure S1.** Alignment of all the sequences of LDH homolog proteins focused in the region corresponding to cluster **B_1_** (**LP22**). The alignment was performed with ClustalO, and conservation was highlighted by shades of blue, from a minimum of 30 % of conservation to a maximum of 70%. Hotspots identified in this study are highly conserved.


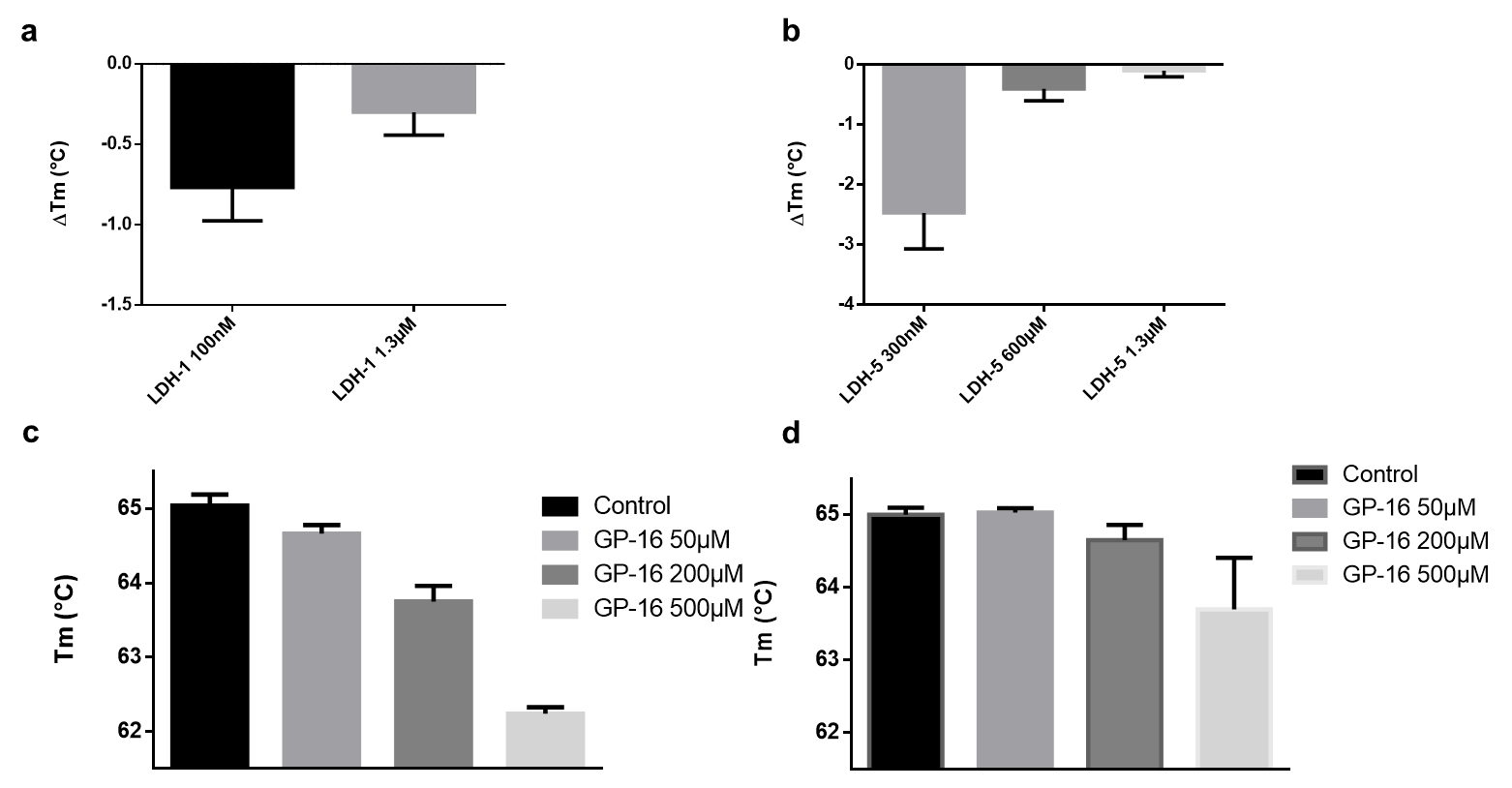


**Figure S2.** a) NanoDSF of LDH-1 at different concentrations when exposed to 500µM of **LP-22**. b) NanoDSF of LDH-5 at different concentrations when exposed to 50µM of **LP-22**. c-d) Thermal stability of LDH-5 at 300nM (c) and 600 nM (d) when exposed to different concentrations of **GP-16**.

**Figure S3.** MST binding curves between **LP-22** and LDH-Htr. Binding curve was extracted from the MST traces at a 1.5 s MST on time (*n = 3*) (K_d_ = 240 µM, CI95% : 188 to 267 µM).


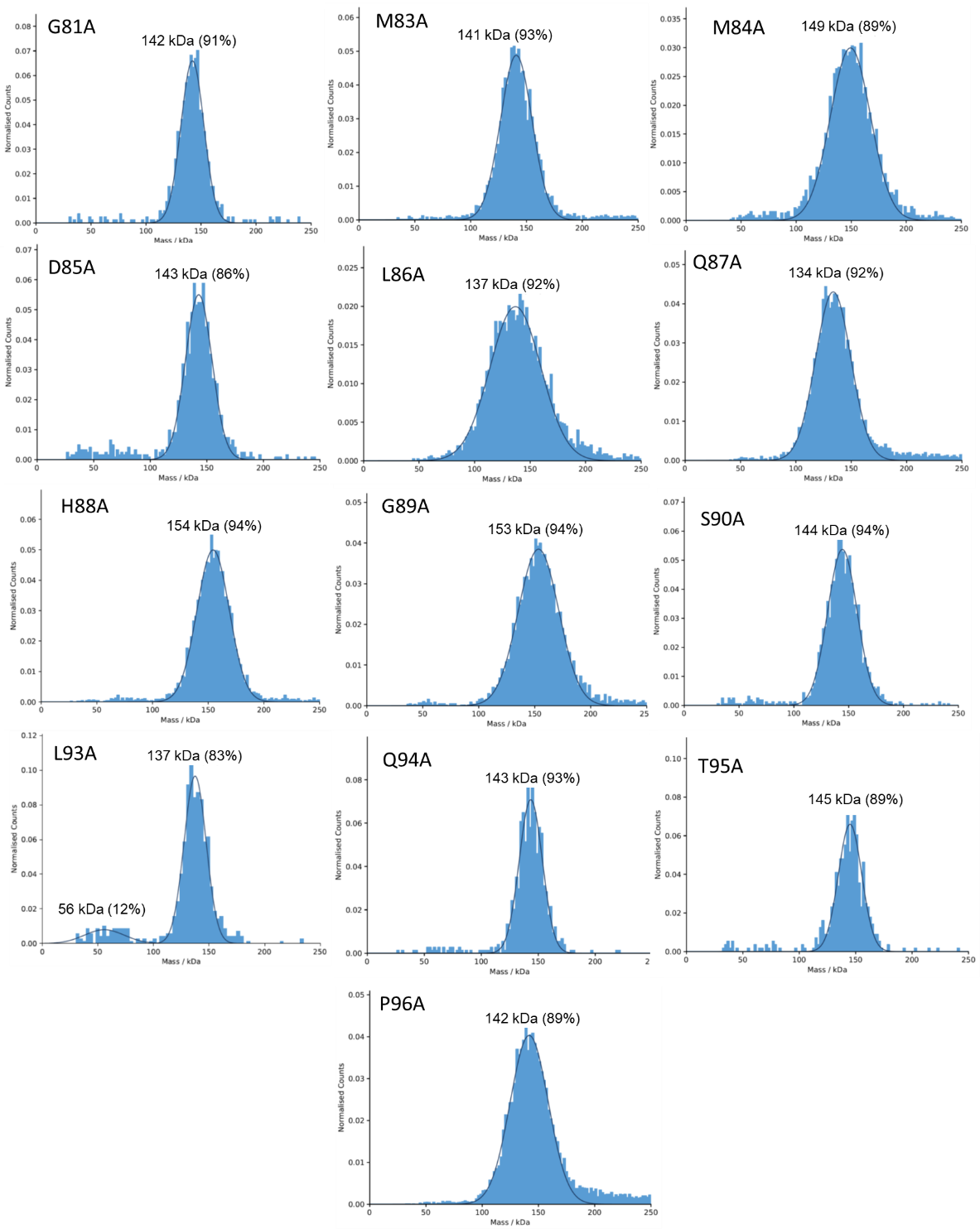


**Figure S4.** Results of the mass photometry experiments performed on different LDH-H variants with the experimental molecular weights of the complexes in solution and their relative intensities. Theoretical molecular weight of the tetramer = 155 kDa; Theoretical molecular weight of the tetramer = 155 kDa; Theoretical molecular weight of the dimer = 78 kDa.
